# Combined Ultrasound and CT-Guided Iodine-125 Seeds Implantation for Treatment of Residual Hepatocellular Carcinoma Located at Complex Sites After Transcatheter Arterial Chemoembolization

**DOI:** 10.3389/fonc.2021.582544

**Published:** 2021-03-02

**Authors:** Yanqiao Ren, Xiangjun Dong, Lei Chen, Tao Sun, Osamah Alwalid, Xuefeng Kan, Yangbo Su, Bin Xiong, Huimin Liang, Chuansheng Zheng, Ping Han

**Affiliations:** ^1^ Department of Radiology, Union Hospital, Tongji Medical College, Huazhong University of Science and Technology, Wuhan, China; ^2^ Hubei Key Laboratory of Molecular Imaging, Wuhan, China

**Keywords:** hepatocellular carcinoma, iodine-125 implantation, complex sites, survival, safety

## Abstract

**Purpose:**

The purpose of this study was to evaluate the efficacy and safety of iodine-125 (^125^I) seeds implantation under ultrasound and computed tomography (CT) guidance in the treatment of residual hepatocellular carcinoma (HCC) located at complex sites after transcatheter arterial chemoembolization (TACE).

**Methods:**

This retrospective study analyzed the consecutive medical records of 31 HCC patients with residual tumors located at complex sites (such as large blood vessels, gallbladder, diaphragm dome, etc.) after TACE from May 2014 to December 2018, all of whom received ^125^I seeds implantation therapy. Overall survival (OS), progression-free survival (PFS), recurrence, and complications were documented.

**Results:**

A total of 607 seeds were implanted in 31 patients, with an average of 19.6±10.4 (range, 8–48) seeds per patient. Median OS and PFS were 33 months (95% CI: 27.1 months, 38.9 months) and 15 months (95% CI: 9.6 months, 20.4 months), respectively. Although univariate analysis showed that albumin, prothrombin time, alpha-fetoprotein level, Child-Pugh score, and lipiodol deposition in tumor were associated with OS, multivariate analysis showed that none of them was an independent prognostic factor for OS. Multivariate analysis showed that prothrombin time was an independent prognostic factor for PFS. No operation-related deaths in this study. Although pneumothorax was present in two patients and subcutaneous abscess in one patient, symptoms improved in all three patients with appropriate treatment. Common minor complications included fever, abdominal pain and leukopenia and no grade≥3 adverse events were observed.

**Conclusions:**

^125^I seeds implantation under the combined guidance of ultrasound and CT is safe and effective for patients with residual HCC located at complex sites after TACE. This is a promising treatment approach and deserves further discussion.

## Introduction

Globally, and especially in China, hepatocellular carcinoma (HCC) remains a challenging and often therapeutically disappointing problem. Although regular ultrasound and serum alpha-fetoprotein (AFP) levels are monitored in high-risk populations, it is reported that 70% of HCC patients are diagnosed in the intermediate Barcelona Clinic Liver Cancer (BCLC) stage or advanced (BCLC stage C) stage (1). Currently, based on two randomized controlled trials (2, 3) and a cumulative meta-analysis (4), transcatheter arterial chemoembolization (TACE) is considered the first-line treatment for patients with intermediate HCC (5). However, some tumor cells may still survive after a session of TACE, with a low complete response (CR) rate ranging from 23% to 27% (6–9), which may be due to the non-dense deposition of iodide oil, leading to incomplete occlusion of the tumor-supplying vessel (6). Therefore, local or intrahepatic residue after TACE is a common clinical problem that needs to be solved urgently.

Clinically, repeated TACE is the most commonly used method for HCC with local or intrahepatic residue (10, 11). At the same time, many studies have shown that the use of yttrium-90 microspheres or iodine-131 lipiodol can improve the prognosis of patients with HCC (12, 13). However, injection of embolizing materials such as yttrium-90 microspheres and iodine-131 lipiodol could not completely inactivate tumors located at complex sites such as diaphragm dome, hepatic hilum and gallbladder. Since the anatomical location of the tumor, adjacent to organs, or previous treatment have been reported as the reasons for the formation of the extrahepatic collaterals, the tumors located at the complex sites are often supplied by the extrahepatic collateral arteries (11). With the current interventional techniques and operation facilities, the extrahepatic collateral arteries are often incompletely embolized, resulting in incomplete tumor necrosis. Although local ablation including percutaneous ethanol injection, radiofrequency ablation (RFA), and microwave ablation have been reported for the treatment of HCC of complex sites, many problems including complex procedures, serious complications, and low rates of complete ablation limit the application of local ablation (14–18). Taken together, effectively treating HCC located at complex sites with residue after TACE is a major challenge in cancer treatment, which may create an incentive to try other therapies and approaches.

Brachytherapy with iodine-125 (^125^I) seeds implantation for high dose irradiation of the lesions has been widely used in the treatment of various cancers including prostate cancer, head and neck malignant neoplasms, pancreatic cancer, and lung cancer (19–23). For HCC patients, many studies have reported that ^125^I seeds implantation can effectively control portal vein tumor thrombus (24–26). In addition, brachytherapy has also been used in the treatment of liver cancer in recent years with good efficacy (27, 28). To the best of our knowledge, there have been no reports on ^125^I seeds implantation for the treatment of residual HCC located at complex sites after TACE. Thus, the objective of this study was to assess efficacy, safety profile and prognostic factors of ^125^I seeds implantation guided by ultrasound and computed tomography (CT) in the treatment of residual HCC located at complex sites after TACE.

## Methods

### Study Design and Patient Selection

The present retrospective, single-center study was conducted in accordance with the principles of the Declaration of Helsinki and all procedures performed in this study were approved by the Ethics Committee of Tongji Medical College, Huazhong University of Science and Technology. Written informed consent was obtained from all patients prior to treatment.

In this retrospective study, we analyzed the electronic medical records of 31 consecutive HCC patients who received ^125^I seeds implantation between May 2014 and December 2018, all of whom had residual tumors located at complex sites after TACE (**Table 1**). HCC located at complex sites refers to tumors adjacent to the gallbladder, diaphragm dome, gastrointestinal tract, heart, large blood vessels, and hepatic hilum, with the shortest distance from the organ or lumen being less than 0.5 cm (14, 29). We investigated 26 males and five females with an average age of 56.0 ± 11.7 (range, 33–73) years old. All patients received at least one session of TACE and had tumor residue, with at least one lesion adjacent to one of the complex sites. Prior to the initial ^125^I seeds implantation, the treatment strategy was determined by the multidisciplinary tumor board. The diagnosis of HCC was based on the diagnostic criteria of the European Association for the Study of Liver and the American Association for the Study of Liver Disease (5, 30). In our study, the diagnosis of HCC was confirmed when two different imaging examinations revealed typical radiographic finding of HCC in a patient with an elevated alpha-fetoprotein level (≥400 ng/ml) or when there was a cytologic or histologic diagnosis of HCC. Inclusion criteria of this study were: (1) residual tumor at complex sites after TACE; (2) age > 18 years old; (3) an Eastern Cooperative Oncology Group (ECOG) performance status of 0 and expected survival of >3 months; (4) Child-Pugh class A or B; (5) no evidence of invasion into the portal or hepatic veins, extrahepatic metastasis, or uncontrolled ascites; (6) patients who refuse to undergo hepatectomy; (7) normal heart, lung and kidney function; (8) white blood cell count ≥4.0X10^9^/L, platelet count ≥50X10^9^/L. Exclusion criteria were: (1) uncontrolled infection; (2) poor performance status (ECOG > 0); (3) hepatic dysfunction (total bilirubin serum >3 mg/dL, serum albumin level <2.0 mg/dL, INR > 1.5), renal impairment (serum creatinine level >2mg/dL).

**Table 1:** Baseline patient characteristics (n = 31).

Characteristic	Patients with ^125^I seeds implantation (No, %; Mean ± SD)
**Gender**	
Male	26 (83.9%)
Female	5 (16.1%)
**Age (y)**	56.0 ±11.7
**Hemoglobin (g/L)**	124 ± 24.8
**Neutrophil (×10^9^/L)**	3.1 ± 2.5
**Platelet count (10^9^/L)**	154.7 ± 42.6
**AST (µmol/L)**	39.4 ± 18.3
**ALT (µmol/L)**	36.5 ± 19.4
**Albumin (g/L)**	37.7 ± 5.7
**Total Bilirubin (µmol/L)**	25.8 ± 16.0
**Creatinine (µmol/L)**	67.7 ± 16.8
**Prothrombin time (s)**	14.4 ± 2.3
**Number of tumors**	
**1**	19 (61.3%)
**>1**	12 (38.7%)
**Ascites**	
Absent	29 (93.5%)
Present	2 (6.5%)
**BCLC stage**	
A	14 (45.2%)
B	17 (54.8%)
**Largest diameter of tumor (cm)**	
≤3	14 (45.2%)
>3	17 (54.8%)
**Hepatitis**	
Hepatitis B	23 (74.2%)
Other	8 (25.8%)
**Alpha-fetoprotein level**	
>400 ng/ml	18 (58.1%)
≤400 ng/ml	13 (41.9%)
**Child-Pugh score**	
A	22 (71.0%)
B **Special site** Large blood vessels Gastrointestinal tract Hepatic hilum	9 (29.0%) 10 (32.3%) 8 (25.8%) 4 (12.9%)
Gallbladder	4 (12.9%)
Diaphragm dome	3 (9.7%)
Heart	2 (6.5%)
**TACE sessions**	
1	12 (38.7%)
2 or more	19 (61.3%)
**Lipiodol deposition in tumor**	
0%–50%	23 (74.2%)
51%–99%	8 (25.8%)

SD, Standard deviation; AST, Aspartate aminotransferase; ALT, Alanine aminotransferase; BCLC, Barcelona Clinic Liver Cancer; TACE, Transcatheter arterial chemoembolization.

## Implantation of ^125^I Seeds

The ^125^I seeds (0.8 mm in diameter and 4.5 mm in length), which emit 27.4–31.5 keV x-ray and 35.5 keV γ-ray with a half-life of 59.6 days, were enclosed in the NiTinol capsule from China Institute of Atomic Energy, Beijing, China. The radioactivity range of each ^125^I seed was 0.5 ~ 0.8 millicuries (mCi), and the tissue penetration depth was 17 mm. One week before the implantation of ^125^I seeds, conventional CT scan was performed on the patients, tumor images of 5 mm thickness were obtained, and the images were transmitted to the Treatment Planning System (TPS; HGGR300, Hokai Medical Instruments Co., Ltd., Zhuhai, China). The number and total activity of the ^125^I seeds to be implanted were determined by TPS based on the minimum peripheral dose (mPD, 90 to 165Gy) per tumor. Therefore, x- and γ-rays can radiate the intended target volume, including the tumor and 0.5–1 cm of the adjacent non-cancerous tissue.

**Figure 1:**
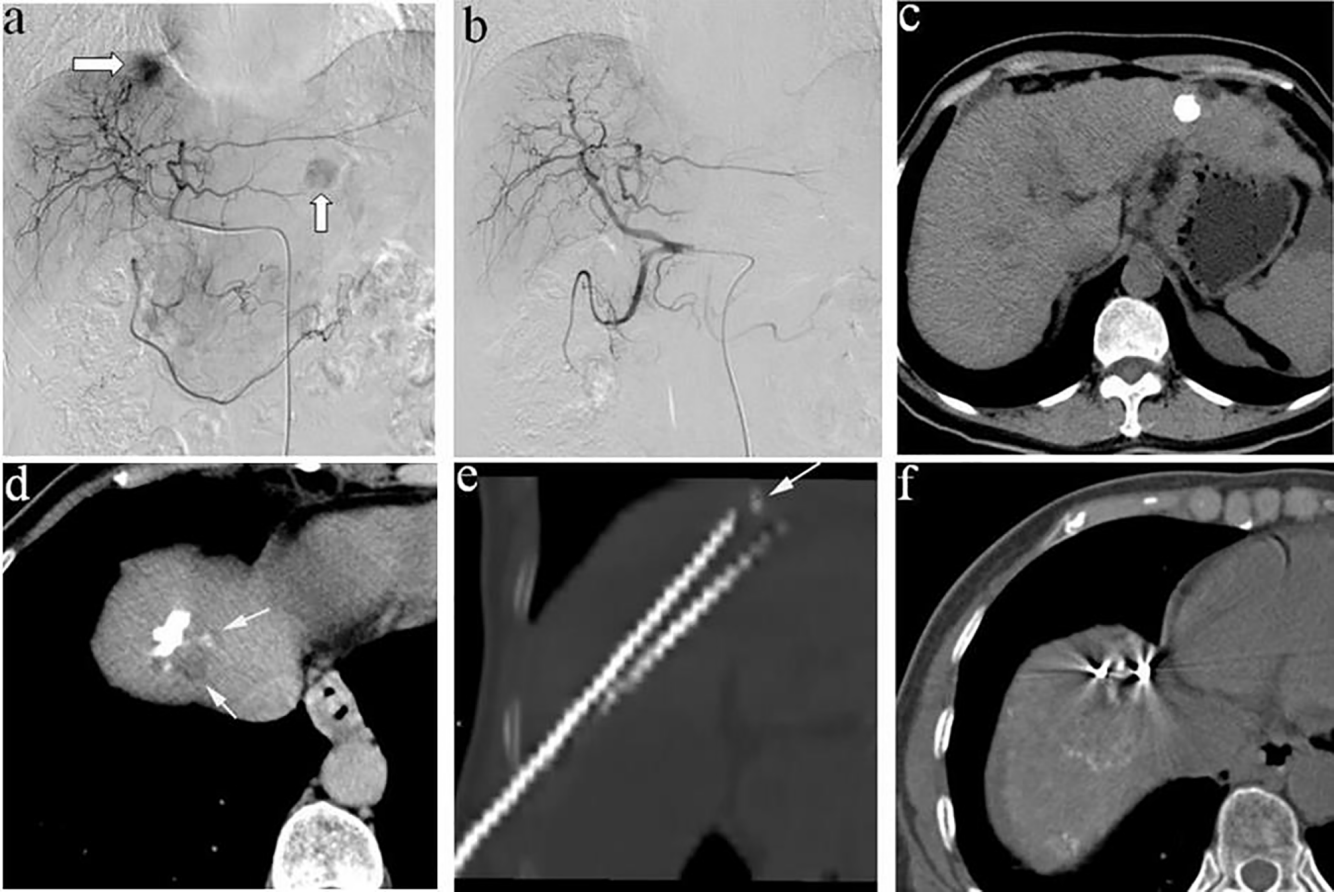
**(A)** A 58-year-old man, angiography revealed two tumors in the liver, one located at the diaphragm dome and the other at the left lobe; **(B)** Complete embolism achieved with TACE; **(C)** CT examination 2 weeks after TACE showed that the lesion located at the left lobe had good iodide deposition, **(D)** while the tumors located at the diaphragm dome had poor iodide deposition and small residual lesions; **(E)** therefore, the patient underwent ^125^I seeds implantation, and **(F)** plain CT scan after ^125^I seeds implantation showed good seeds distribution.

^125^I seeds implantation was performed by two experienced interventional radiologists (HML and CSZ, with more than 10 years of experience in ^125^I seeds implantation) under local anesthesia using 5% lidocaine (**Figure 1**). Patients have been instructed to breathe calmly during the procedure. Under the dual guidance of ultrasound and CT, 18 G needles and a turntable implantation gun (XinKe Pharmaceutical Ltd., Shanghai, China) were used to implant the temperature-sterilized seeds into the tumor at the interval of 1cm. CT examination was performed again after implantation of ^125^I seeds to observe the distribution of seeds and ensure their uniform distribution within the tumor. After the procedure, antibiotics were given to prevent infection and analgesics and hemostatic drugs were used according to the patient’s condition.

### Definition and Data Evaluation

Overall survival (OS) was defined as the interval between the first ^125^I seeds implantation and the date of death or last follow-up. Progression-free survival (PFS) referred to the period between the date of the initial ^125^I seeds implantation and the date of progression for patients who displayed radiologic evidence of disease progression or the date of death. In this study, tumor recurrence was divided into local recurrence, intrahepatic recurrence and extrahepatic metastasis. Local recurrence was defined as the presence of tumor staining within or at the periphery of the original ^125^I seed implantation site. Intrahepatic recurrence refers to the appearance of new liver lesions more than 2.0 cm away from the primary lesion. Extrahepatic metastasis was defined as an extrahepatic tumor lesion.

We used the Society of Interventional Radiology classification system to assess the safety of HCC patients receiving ^125^I seeds implantation (31). Major complications referred to events leading to death or disability that increase the level of care, or result in hospital admission, or substantially lengthen the hospital stay. Complications such as fever, abdominal pain, and liver dysfunction were considered minor. Meanwhile, the Common Terminology Criteria for Adverse Events (version 5.0) was also used to evaluate complications or side effects.

### Follow-Up

All the patients were followed up until January 30, 2020. Patients were evaluated 1 month after the initial ^125^I seeds implantation. The evaluation included laboratory tests (hematology and biochemical markers) and abdominal contrast-enhanced CT or magnetic resonance imaging (MRI). Repeat ^125^I seeds implantation or TACE were performed if residual viable HCCs or recurrent tumors were documented by contrast-enhanced CT or MR and the patient had preserved liver function. If no residual or recurrent tumor was shown, contrast-enhanced CT or MR and laboratory tests (including blood routine, liver, and kidney function, AFP level, coagulation function, etc.) were performed every 3 months. Follow-up continued until the patient died or until January 30, 2020.

### Statistical Analyses

All analyses were performed using SPSS software (Version 24.0; IBM, Armonk, New York), and *P* < 0.05 indicated a statistically significance. Discrete variables were presented as numbers with percentages, and continuous variables were presented as mean ± standard deviation. OS and PFS were calculated by using Kaplan-Meier method. The 95% confidence interval (CI) was calculated for median OS, median PFS, and hazard ratio (HR). The log-rank test was used for univariate analyses. Variables with a value of *P* < 0.10 at univariate analysis were added to multivariate analysis. A Cox proportional hazard regression model was used to analyze the potential prognostic factors affecting OS and PFS.

## Results

### Study Population

During the study period, a total of 31 HCC patients received ^125^I seeds implantation, with an average of 1.2±0.4 (range, 1-2) ^125^I seeds implantation procedure per patient. A total of 607 seeds were implanted, with an average of 19.6±10.4 (range, 8–48) per patient. There were 10, 8, 4, 4, 3, and 2 patients with residual tumors adjacent to the large blood vessels, gastrointestinal tract, hepatic hilum, gallbladder, diaphragm dome, and heart, respectively. Mean tumor diameter before seeds implantation was 3.3±1.6 cm (range, 1–6.6 cm). TACE was performed in an average of 2.3±1.4 times per patient (range, 1–6 times) before seeds implantation. The median follow-up period in this study was 29 months (range 9–67 months). At the end of follow-up (January 30, 2020), 20 (64.5%) patients died during the observation period.

### Overall Survival

**Figure 2:**
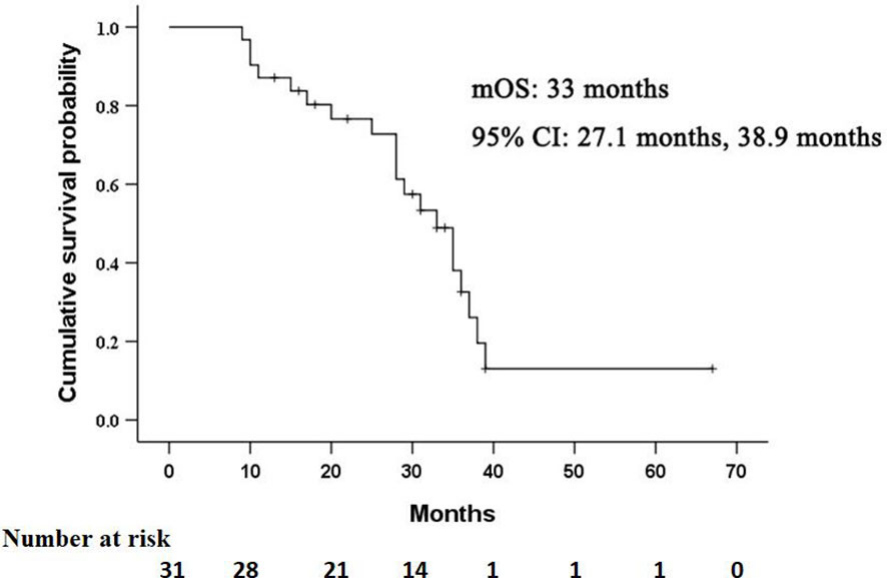
Kaplan–Meier curves of overall survival (OS) in patients with residual hepatocellular carcinoma located at complex sites after transcatheter arterial chemoembolization.

The median OS in this study was 33 months (95% CI: 27.1 months, 38.9 months), as shown in **Figure 2**. Univariate analysis revealed that albumin, prothrombin time, AFP level, Child-Pugh score, and lipiodol deposition in tumor were correlated with OS (**Table 2**), but when these five variables were included in the multivariate analysis (**Table 3**), the results showed that none of them was an independent prognostic factor for OS (*P* > 0.05).

**Table 2:** Univariate analysis of prognostic factors for overall survival and progression-free survival.

Variables	OS		PFS	
	HR (95% CI)	*P* value	HR (95% CI)	*P* value
**Gender**				
Male	1		1	
Female	0.343 (0.045, 2.589)	0.299	0.279 (0.066, 1.191)	**0.085**
**Age (y)**	1.009 (0.965, 1.054)	0.704	1.010 (0.973, 1.048)	0.600
**Hemoglobin (g/L)**	1.021 (0.996, 1.047)	0.105	1.026 (1.008, 1.045)	**0.005**
**Neutrophil (×10^9^/L)**	1.067 (0.923, 1.234)	0.380	1.101 (0.973, 1.246)	0.129
**Platelet count (10^9^/L)**	1.001 (0.989, 1.014)	0.836	1.000 (0.990, 1.010)	0.990
**AST (µmol/L)**	1.007 (0.983, 1.031)	0.593	0.996 (0.973, 1.020)	0.735
**ALT (µmol/L)**	1.007 (0.989, 1.024)	0.470	1.008 (0.990, 1.027)	0.367
**Albumin (g/L)**	1.088 (0.985, 1.202)	**0.097**	1.116 (1.024, 1.218)	**0.013**
**Total Bilirubin (µmol/L)**	0.996 (0.967, 1.025)	0.765	0.987 (0.959, 1.017)	0.406
**Creatinine (µmol/L)**	1.013 (0.991, 1.036)	0.252	1.023 (1.000, 1.048)	**0.053**
**Prothrombin time (s)**	0.772 (0.575, 1.035)	**0.083**	0.669 (0.501, 0.894)	**0.007**
**Number of tumors**				
>1	1		1	
1	1.188 (0.482, 2.930)	0.709	1.089 (0.482, 2.462)	0.838
**Ascites**				
Present	1		1	
Absent	0.942 (0.123, 7.216)	0.954	2.181 (0.293, 16.221)	0.446
**BCLC stage**				
B	1		1	
A	0.784 (0.318, 1.936)	0.598	0.675 (0.304, 1.499)	0.334
**Largest diameter of tumor (cm)**				
≤3	1		1	
>3	1.275 (0.517, 3.146)	0.598	1.481 (0.667, 3.290)	0.334
**Hepatitis**				
Hepatitis B	1		1	
Other	1.413 (0.554, 3.603)	0.469	1.334 (0.573, 3.108)	0.504
**Alpha-fetoprotein level**				
>400 ng/ml	1		1	
≤400 ng/ml	0.321 (0.122, 0.844)	**0.021**	0.403 (0.173, 0.940)	**0.035**
**Child-Pugh score**				
B	1		1	
A	0.376 (0.139, 1.015)	**0.053**	0.542 (0.238, 1.234)	0.145
**TACE sessions**				
1	1		1	
2 or more	1.005 (0.389, 2.594)	0.992	1.812 (0.793, 4.140)	0.159
**Lipiodol deposition in tumor**				
51%-99%	1		1	
0%-50%	2.541 (0.899, 7.182)	**0.079**	0.716 (0.298, 1.718)	0.454

OS, Overall survival; PFS, Progression-free survival; HR, Hazard ratio; CI, Confidence interval; AST, Aspartate aminotransferase; ALT, Alanine aminotransferase; BCLC, Barcelona Clinic Liver Cancer; TACE, Transcatheter arterial chemoembolization. Values in bold represent values with P value < 0.1.

**Table 3:** Multivariate analysis of prognostic factors for overall survival.

Variables	HR (95% CI)	*P* value
**Albumin (g/L)**	1.042 (0.927, 1.172)	0.487
**Prothrombin time (s)**	0.821 (0.590, 1.142)	0.241
**Alpha-fetoprotein level**		
>400 ng/mL	1	
≤400 ng/ml	0.382 (0.129, 1.128)	0.081
**Child-Pugh score**		
B	1	
A	0.456 (0.151, 1.372)	0.163
**Lipiodol deposition in tumor**		
51%-99%	1	
0%-50%	1.042 (0.927, 1.172)	0.991

HR, Hazard ratio; CI, Confidence interval.

### Progression-Free Survival and Recurrence

**Figure 3:**
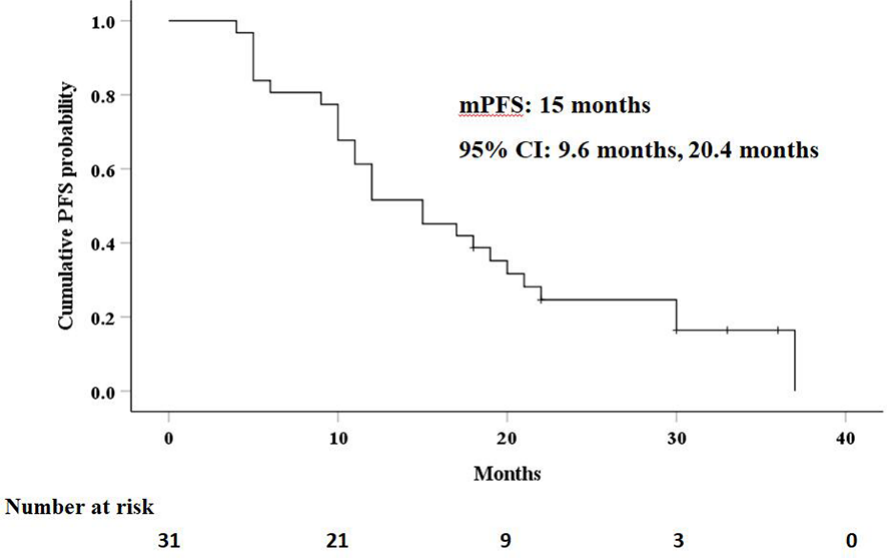
Kaplan–Meier curves of progression-free survival (PFS) in patients with residual hepatocellular carcinoma located at complex sites after transcatheter arterial chemoembolization.

The median PFS in this study was 15 months (95% CI: 9.6 months, 20.4 months), as shown in **Figure 3**. Univariate analysis (**Table 2**) indicated that gender, hemoglobin, albumin, creatinine, prothrombin time, and AFP level were related to PFS. On this basis, these six factors were included in a multivariate analysis, and prothrombin time was identified as an independent prognostic factor for PFS (**Table 4**). A total of 24 patients (77.4%) had recurrence during the follow-up period, including three patients (9.7%) with local recurrence, 17 patients (54.8%) with intrahepatic recurrence, and four patients (12.9%) with extrahepatic metastases.

**Table 4:** Multivariate analysis of prognostic factors for progression-free survival.

Variables	HR (95% CI)	*P* value
**Gender**		
Male	1	
Female	0.307 (0.057, 1.652)	0.169
**Hemoglobin (g/L)**	1.016 (0.993, 1.041)	0.174
**Albumin (g/L)**	0.978 (0.884, 1.082)	0.667
**Creatinine (µmol/L)**	1.018 (0.989, 1.047)	0.235
**Prothrombin time (s)**	0.583 (0.384, 0.885)	0.011
**Alpha-fetoprotein level**		
>400 ng/ml	1	
≤400 ng/ml	0.675 (0.256, 1.775)	0.425

HR, Hazard ratio; CI, Confidence interval.

### Complications

No procedure-related mortalities occurred. Two patients developed mild pneumothorax, which improved after conservative treatment. Subcutaneous abscess occurred in 1 patient after ^125^I seeds implantation, which disappeared after drainage and antibiotic treatment. During follow-up, no ^125^I seeds were seen to migrate from the liver to other organs such as the lungs or heart. Common minor complications occurred in 11 patients (35.5%), including fever in nine patients (29.0%), abdominal pain in five patients (16.1%), nausea and vomiting in four patients (12.9%), and leukopenia in six patients (19.4%). As summarized in **Table 5**, no grade≥3 adverse events were observed. These symptoms lasted 2–7 days and then improve gradually.

**Table 5:** Adverse events related to TACE and ^125^I seeds implantation.

Adverse Event	All Events	CTCAE Grade		
		1	2	≥3
**Fever**	9 (29.0%)	5 (16.1%)	4 (12.9%)	0 (0%)
**Abdominal pain**	5 (16.1%)	2 (6.5%)	3 (9.7%)	0 (0%)
**Nausea and vomiting**	4 (12.9%)	1 (3.2%)	3 (9.7%)	0 (0%)
**Leukopenia**	6 (19.4%)	4 (12.9%)	2 (6.5%)	0 (0%)
**Pneumothorax**	2 (6.5%)	2 (6.5%)	0 (0%)	0 (0%)
**Subcutaneous abscess**	1 (3.2%)	0 (0%)	1 (3.2%)	0 (0%)
**Seeds migration**	0 (0%)	0 (0%)	0 (0%)	0 (0%)
**Total**	27 (87.1%)	14 (45.2%)	13 (41.9%)	0 (0%)

TACE, Transcatheter arterial chemoembolization; CTCAE, Common Terminology Criteria for Adverse Events.

## Discussion

Currently, the treatment of residual HCC adjacent to the gallbladder, diaphragm dome, gastrointestinal tract, heart, large blood vessels, and hepatic hilum remains a challenge. Aggressive surgical resection offers the potential for cure, but for HCC located at complex sites, in addition to considering the patient’s liver function and clinical condition, the inability to obtain adequate surgical margin also limits the application of surgery (19, 28). RFA is a safe and effective treatment for HCC. However, studies have shown that RFA is difficult to achieve complete ablation for tumors located at complex sites, and recurrence is common (32–34). The application of TACE is also limited due to the extrahepatic collateral arteries and the non-dense deposition of iodide oil (6, 11).

It has been reported that ^125^I brachytherapy can achieve high locoregional tumors control (35). The study by Chen et al. (27) showed that compared with RFA alone, RFA combined with ^125^I seeds implantation could significantly improve OS and cumulative recurrence in patients with small HCCs. Yet, no reports on residual HCC located at complex sites after TACE treated with ^125^I seeds implantation. Therefore, this study mainly evaluated the efficacy and safety of ^125^I seeds implantation in these patients.

In the present study, the median OS and PFS were 33 months and 15 months, respectively. Similarly, a retrospective analysis of clinical data of 18 patients with HCC located beneath the diaphragm was conducted. TACE combined with ^125^I seeds implantation was used in these patients, and the results showed that seeds implantation was safe and effective (6). Li et al. (36) suggested that for inoperable 3–5 cm HCC, patients treated with ^125^I seeds implantation combined with TACE had better OS than patients treated with TACE alone. In this study, only three patients experienced local recurrence, with a local recurrence rate of 9.7%. The study of Chen et al. (27) demonstrated that the local recurrence rate of patients in the RFA combined with ^125^I seeds implantation group was significantly lower than that of patients in the RFA alone group (*P*=0.012). At the same time, another randomized controlled trial (37) also indicated that ^125^I seeds implantation can reduce the recurrence rate of HCC after complete hepatectomy. This indicates that ^125^I seeds implantation, as described by the theoretical advantages, may be an effective and feasible option for residual HCC after TACE to provide better tumor control.

A total of 607 seeds were implanted in this study, with an average of 19.6±10.4 per patient and ^125^I seeds were implanted under the dual guidance of ultrasound and CT. The 18G puncture needle can quickly enter the target lesions under the guidance of ultrasound, and CT can accurately locate the position of the needle tip. Due to the influence of patients’ non-consistent respiratory movement, CT guidance alone needed to adjust the puncture angle and path repeatedly. Dual guidance with ultrasound and CT can also reduce the number of CT scans and X-ray exposure time. Lin et al. (19) performed ^125^I seeds implantation under MRI guidance for the treatment of HCC adjacent to large blood vessels, which they considered to be a safe and effective approach. However, the high cost and long MRI-guided operation time limit its application.

The safety of ^125^I seeds in the treatment of HCC is also of a current concern, and safety is particularly important in the treatment of tumors adjacent to the gallbladder, diaphragm dome, gastrointestinal tract, heart, large blood vessels, and hepatic hilum. Similar to other studies (6, 38), there were no operation-related deaths in this study. In addition, although pneumothorax was present in two patients and subcutaneous abscess in one patient, symptoms improved in all three patients with appropriate treatment. Meanwhile, the seeds also did not migrate to other organs during follow-up. Common minor complications in this study included fever, abdominal pain, and leukopenia, most of which were self-limited.

This study was retrospectively designed and has certain limitations. This case series analyzed the efficacy and safety of ^125^I for residual HCC located at complex sites after TACE, but did not compare with other treatment modalities such as repeated TACE or RFA. Therefore, future prospective studies are necessary to further verify the efficacy of ^125^I for complex sites HCCs.

## Conclusion

The treatment of residual HCC adjacent to complex sites after TACE is challenging, and no good therapeutic methods have been reported so far. This study demonstrated that ^125^I seeds implantation under combined ultrasound and CT guidance had good efficacy and was safe enough for the patients. Therefore, for HCC located at complex sites, seeds implantation may achieve satisfactory results, which, of course, needs to be verified by a large number of prospective randomized controlled trials in the future.

## Data Availability Statement

The raw data supporting the conclusions of this article will be made available by the authors, without undue reservation.

## Ethics Statement

The studies involving human participants were reviewed and approved by the Ethics Committee of the Tongji Medical college, Huazhong University of Science and Technology. The patients/participants provided their written informed consent to participate in this study.

## Author Contributions

YR, XD, CZ, and PH conceptualized the study. YR and XD developed the methodology. XD, LC, TS, and OA validated the study. YR, XD, LC, TS, OA, XK, YS, BX, CZ, and PH performed the formal analysis. YR wrote and prepared the original draft. YR, XD, OA, CZ, and PH wrote, reviewed, and edited the article. YR and XD conducted the visualization. XK, YS, BX, and HL supervised the study. All authors contributed to the article and approved the submitted version.

## Funding

This study was funded by grant from the National Science Foundation of China (81873919).

## Conflict of Interest

The authors declare that the research was conducted in the absence of any commercial or financial relationships that could be construed as a potential conflict of interest.

## Abbreviations

HCC, Hepatocellular carcinoma; AFP, Alpha-fetoprotein; BCLC, Barcelona Clinic Liver Cancer; TACE, Transcatheter arterial chemoembolization; CR, Complete response; RFA, Radiofrequency ablation; ^125^I, Iodine-125; ECOG, Eastern Cooperative Oncology Group; CT, Computed tomography; OS, Overall survival; PFS, Progression-free survival; mRECIST, Modified Response Evaluation Criteria in Solid Tumors; PR, Partial response; PD, Progressive disease; SD, Stable disease; ORR, Objective tumor radiologic regression; DCR, Disease control rate; MRI, magnetic resonance imaging; CI, Confidence interval; HR, Hazard ratio.
